# Micronuclei, Pesticides, and Element Mixtures in Mining Contexts: The Hormetic Effect of Selenium

**DOI:** 10.3390/toxics11100821

**Published:** 2023-09-29

**Authors:** Marcela E. Varona-Uribe, Sonia M. Díaz, Ruth-Marien Palma, Leonardo Briceño-Ayala, Carlos Trillos-Peña, Eliana M. Téllez-Avila, Lyda Espitia-Pérez, Karina Pastor-Sierra, Pedro Juan Espitia-Pérez, Alvaro J. Idrovo

**Affiliations:** 1 School of Medicine and Health Sciences, Universidad del Rosario, Bogotá D.C. 111221, Colombia; marcela.varona@urosario.edu.co (M.E.V.-U.); sdiaz21@gmail.com (S.M.D.); leonardo.briceno@urosario.edu.co (L.B.-A.); carlos.trillos@urosario.edu.co (C.T.-P.); 2 Environmental and Occupational Health Group, National Institute of Health, Bogotá D.C. 111321, Colombia; rpalma@ins.gov.co (R.-M.P.); etellez@ins.gov.co (E.M.T.-A.); 3 Grupo de Investigación Biomédicas y Biología Molecular, Universidad del Sinú, Montería 230001, Colombia; lydaespitia@unisinu.edu.co (L.E.-P.); karinapastor@unisinu.edu.co (K.P.-S.); pedrojespitia@unisinu.edu.co (P.J.E.-P.); 4 Public Health Department, School of Medicine, Universidad Industrial de Santander, Bucaramanga 680002, Colombia

**Keywords:** pesticides, mining, chemical mixtures, selenium, hormesis, genetic damage, Colombia

## Abstract

The contexts where there are mining and agriculture activities are potential sources of risk to human health due to contamination by chemical mixtures. These contexts are frequent in several Colombian regions. This study explored the potential association between the frequency of micronuclei and pesticides and elements in regions with ferronickel (Montelibano, Córdoba) and gold (Nechí, Antioquia) mining, and a closed native mercury mine (Aranzazu, Caldas), with an emphasis in the potential effect of selenium as a potential chelator. A cross-sectional study was carried out with 247 individuals. Sociodemographic, occupational, and toxicological variables were ascertained. Blood and urine samples were taken for pesticide analysis (5 organophosphates, 4 organochlorines, and 3 carbamates), 68 elements were quantified in hair, and micronuclei were quantified in lymphocytes. The mixtures of elements were grouped through principal component analysis. Prevalence ratios were estimated with robust variance Poisson regressions to explore associations. Interactions of selenium with toxic elements were explored. The highest concentrations of elements were in the active mines. The potentially most toxic chemical mixture was observed in the ferronickel mine. Pesticides were detected in a low proportion of participants (<2.5%), except paraoxon-methyl in blood (27.55%) in Montelibano and paraoxon-ethyl in blood (18.81%) in Aranzazu. The frequency of micronuclei was similar in the three mining contexts, with means between 4 to 7 (*p* = 0.1298). There was great heterogeneity in the exposure to pesticides and elements. The “hormetic effect” of selenium was described, in which, at low doses, it acts as a chelator in Montelibano and Aranzazu, and at high doses, it can enhance the toxic effects of other elements, maybe as in Nechí. Selenium can serve as a protective agent, but it requires adaptation to the available concentrations in each region to avoid its toxic effects.

## 1. Introduction

Pesticides are chemical substances used to prevent or control any pest, and their inadequate handling can have adverse effects on the health of agricultural workers, their families, the community, and the environment [1]. Exposure to these substances can be through skin contact, ingestion, or inhalation and can cause acute or chronic poisoning. Chronic adverse effects usually appear long after the first exposure, even at low doses, being more frequent among adults [2] since various pesticides accumulate in the body and the environment, causing disease after several years of exposure [3]. There is evidence of the relationship between chronic exposure to some pesticides and an increased occurrence of neurological, respiratory, dermatological, and renal diseases, reproductive disorders, cancer, and endocrine disorders, among others. Many of these disorders are associated with insecticides and herbicides, especially organophosphates, organochlorines, and triazine compounds [4,5]. Considering the availability of susceptibility biomarkers, carcinogenic effects of pesticides can be seen in early changes in genomic DNA, such as DNA-DNA and DNA-protein cross-linking, strand breaks, and DNA adduct formation, generating defective cells [6,7,8,9]. Moreover, the study of micronuclei has been useful in identifying agents associated with inflammation, obesity, adverse reproductive outcomes (infertility and pregnancy complications), chronic diseases (diabetes mellitus, cardiovascular, kidney, and neurodegenerative diseases), and accelerated aging syndromes [10].

Agriculture for human and animal consumption is also practiced in many places where mining activities are performed. The consequences of mining and agricultural activity in the same place are not very well known, but they undoubtedly have to do with the type of mining, the geochemical of soil, and the absorption capacity of crops. Open-pit mining is often associated with extensive environmental damage, as mining waste can spread via air and surrounding water sources. It contrasts with damage to water sources related to alluvial and underground mining, although it has higher deleterious effects in alluvial activities [6,11]. Concerning the geochemistry of soils and types of vegetation, generalizations are not easy since the chemical mixtures observed in rocks and soils can be highly variable. For this, specific studies are required for each mining-agricultural region. Research on exposure to pesticides, elements, and genetic damage has been explored in several studies, but mainly in isolation from other chemicals. Studies on mixtures of pesticides with toxic substances, trace elements, and substances with no known adverse effects in humans, animals, and plants are scarce, even though it is a common form of exposure.

In Colombia, there is a high consumption and market of pesticides, even though the records are inaccurate [12]. In this country, mining regions may coincide with crops cultivated years ago or fumigated to prevent vector-borne transmitted diseases [13]. When pesticides are persistent in the environment, such as organochlorines, the presence of toxic minerals may propitiate the occurrence of adverse effects not well-known in humans [14]. Although organochlorine pesticides have been forbidden in Colombia, there is evidence that they were used to control pests in coca, marijuana, and poppy crop regions [15]. In this regard, there are approximately 88,629 m^3^ of soil contaminated with organochlorines in the country; however, there is a significant underreporting of contaminated sites [16].

The legal consumption of pesticides in Colombia includes agricultural and livestock uses, the control of vector-borne diseases, and the use of glyphosate in the illicit crop control program. It is estimated that ~13.17 kg of pesticide per hectare (kg/ha) are used in the country, much higher than the world average estimated at 2.57 kg/ha [17]. The largest amounts of pesticides are used in agriculture, where organophosphates, carbamates, dithiocarbamates, phosphonic acid, pyrethroids, and bipyridyls are more frequent. Pyrethroids and organophosphates with residual action are used to control vector-borne diseases in the places with the highest occurrence [18]. Glyphosate has been used in the illicit crop control program in two commercial presentations: Cuspide^®^ 480SL has been applied for ground spraying and Roundup-Ultra^®^ (glyphosate + Cosmoflux 411F^®^) for aerial spraying [19]. The exposure to glyphosate in the control of illicit crops is much higher than that observed in other uses around the world [20,21]. In addition, it should be noted that, in 2015, the use of glyphosate in these activities was abandoned due to the potential carcinogenic risk [22], but it continues to be used in other commercial presentations for agricultural uses.

In these mining-agricultural contexts, multiple and heterogeneous exposures can generate synergistic effects; that lead to greater damage or disease or antagonistic effects where the adverse effects of exposure are minimized by the presence of other chemical compounds. Perhaps the most notorious case in Colombia may be the presence of very high selenium concentrations in soils [23], which may reduce the adverse effects of metals and metalloids given their chelating action [24]. However, the presence of recognized toxics, such as mercury, lead, cadmium, and pesticides, with elements that are not widely studied in the country, such as arsenic, beryllium, and uranium [25,26], could have unpredictable effects. Potential adverse effects in humans due to exposure to complex mixtures of chemical substances are chronic diseases [7,8] and genomic damage [27,28]. In this context, this study explored the association between exposure to some pesticides and elements related to anthropogenic and non-anthropogenic activities with the occurrence of micronuclei among individuals residing in three mining areas in Colombia. Potential selenium effects were emphasized. It corresponds to a first approximation of potential adverse effects among humans in mining contaminated sites with agricultural or vector-borne disease control activities.

## 2. Materials and Methods

### 2.1. Study Design and Mining Contexts

**Figure 1:**
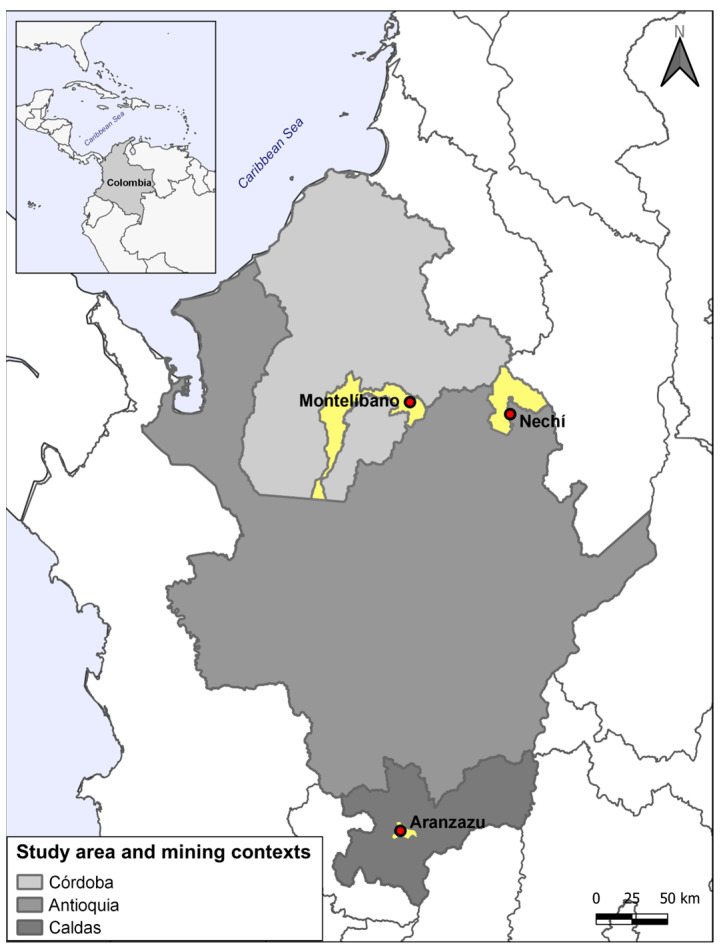
Mining contexts with agricultural activities included in the study.

A cross-sectional study was conducted with three populations potentially exposed to mining and agricultural activities. The populations were from Montelibano (Córdoba), Nechí (Antioquia), and Aranzazu (Caldas) (see Figure 1).

The selection of these municipalities was based on the specific characteristics of each place. Montelibano is close to one of the largest ferronickel mines in the world. Additionally, there is a coal mine in the region, and gold is exploited in an artisanal way. Exposure to chemical mixtures (Ni, Fe, As, Pb, Cd, mainly) has been described [29,30]. In this region, *Plasmodium falciparum* infection is frequent [31]; thus, insecticides are used. Moreover, cassava and purple yam (*Dioscorea alata*) are common crops in this territory. Nechí is a municipality where gold is extracted from alluvium, which has led to the use of mercury for decades to separate and extract the precious metal from other rocks. According to previous studies, it is one of the most mercury-contaminated areas in the world. Mercury levels in the air are from 300 to 1 million ng Hg/m^3^ in public spaces and ~10,000 ng Hg/m^3^ in private domestic sites [32]. Moreover, in this region, pesticides are used in crops of rice, corn, cassava, banana, and purple yam and to control vector-borne diseases. In Aranzazu, there is a geological fault where native mercury presence is important; in fact, the only mercury mine in Colombia exists in this municipality. It was closed in 1975 due to financial problems and neurotoxicity among the workers [33]. Currently, it is a small municipality whose economic activities are related to avocado and coffee cultivation. It is an example of what can happen after a mercury mine is closed.

### 2.2. Participants and Data Collection

Potential participants from the three municipalities were selected by non-probabilistic sampling due to the lack of an updated sampling frame and the logistical limitations related to informal work and difficult access to mining regions. In the Nechí and Montelibano regions, for decades, there have been illegal armed groups, which has limited the presence of public and environmental health researchers due to the inherent security risks. Individuals ≥18 years old living in the place for a minimum of six months participated in the study. Those individuals who had presented illnesses that prevented them from adequately answering the questionnaire and who had undergone X-rays, radiotherapy, or chemotherapy during the previous year were excluded. Consequently, participants were formal and informal workers or individuals dedicated to housework activities. A questionnaire was applied that included sociodemographic variables (age, sex, education, health affiliation), labor, and toxicological (smoker status, alcohol consumption). Additionally, an adaptation of the World Health Organization’s instrument, “Guidance for identifying populations at risk from mercury exposure”, was applied [34].

### 2.3. Pesticides in Blood and Urine

A 5 mL venous blood sample and a 50 mL urine sample were extracted from each participant, which remained refrigerated until processing and analysis to determine pesticides. Concentrations (ppb) of carbamate insecticides: aldicarb, carbofuran, and propoxur; the organochlorines: α-endosulfan, β-endosulfan, endosulfan sulfate, and hexachlorobenzene, and the organophosphorus: malathion, ethyl paraoxon, methyl paraoxon, ethyl parathion, and methyl parathion, were determined in blood and urine. They were selected because they are widely used in Colombia and have high acute toxicity. The analyses were performed through Quick, Easy, Cheap, Effective, Rugged, and Safe (QuEChERS) extraction and high-performance liquid chromatography (HPLC) analyses with triple quadrupole (SQ) LC/MSD. We rely on the method published by Sciex (equipment supplier) for environmental applications [35]. The limits of detection are available in Appendix A (Table A1 and Table A2).

### 2.4. Element Mixtures in Hair

Hair (in high proportion keratin rich in sulfur) was selected as an internal dose biomarker given the interest in quantifying metals and metalloids; the way of entering the body is via ingestion, although the concentration of some elements may be due to external contamination or compounds included in cosmetics [36]. Hair samples were taken from the occipital region of the scalp (~3 cm long) of all participants. The 68 elements included in the analysis were: lithium (Li), beryllium (Be), boron (B), sodium (Na), magnesium (Mg), aluminum (Al), silicon (Si), phosphorus (P), sulfur (S), potassium (K), calcium (Ca), scandium (Sc), titanium (Ti), vanadium (V), chromium (Cr), manganese (Mn), iron (Fe), cobalt (Co), nickel (Ni), copper (Cu), zinc (Zn), gallium (Ga), germanium (Ge), arsenic (As), bromine (Br), selenium (Se), rubidium (Rb), strontium (Sr), yttrium (Y), zirconium (Zr), niobium (Nb), molybdenum (Mo), ruthenium (Ru), rhodium (Rh), palladium (Pd), silver (Ag), cadmium (Cd), indium (In), tin (Sn), antimony (Sb), iodine (I), cesium (Cs), barium (Ba), lanthanum (La), cerium (Ce), praseodymium (Pr), neodymium (Nd), samarium (Sm), europium (Eu), gadolinium (Gd), terbium (Tb), dysprosium (Dy), holmium (Ho), erbium (Er), thulium (Tm), ytterbium (Yb), lutetium (Lu), tantalum (Ta), tungsten (W), rhenium (Re), platinum (Pt), gold (Au), mercury (Hg), thallium (Tl), lead (Pb), bismuth (Bi), thorium (Th), and uranium (U). Note that several elements (or related compounds) listed by IARC as carcinogenic (group 1), probable (2A), or possible (2B) carcinogenic to humans were included in the analysis. Group 1 are As, Be, Cd, Cr, Sr, Ni, and Th. Group 2A are Sb, Co, In, Mg, Mn, and Si. Group 2B are Hg, Mo, Pb, K, Na, Ti, and V.

The preparation and the sample analysis were carried out by the ICP-MS and ICP OES Laboratory (LABSPECTRO) of the Chemistry Department of the Pontificia Universidade Católica do Rio de Janeiro, in Brazil. Over there, the samples were cleaned with deionized water and acetone for three repetitions, with each solution inside an ultrasound bath to eliminate exogenous elements. Hair samples were oven-dried at 60 °C and weighed. Samples were then acidified with 2.50 mL of HNO_3_ in 50 mL polypropylene tubes and digested at 70 °C for 4 h. Finally, they were diluted for elemental determination. Hair element concentrations were evaluated by inductively coupled plasma mass spectrometry using an Elan DRCII ICP-MS spectrometer (Analyst 200, PerkinElmer, Sciex, Norwalk, CT, USA) [37]. Each curve calibration point (blank, reagent blank, and sample) was analyzed with the internal rhodium standard. Argon was used as carrier gas at a flow rate of 50 mL min^−1^.

### 2.5. Cytogenetic Analysis

Fenech’s methodology was used to explore genetic instability [38]. First, heparinized whole blood (0.5 mL) was added to 4.5 mL of RPMI 1640 medium (Sigma R8758, St. Louis, MO, USA) supplemented with 2 mM L-glutamine (Sigma A5955, St. Louis, MO, USA), 10% fetal bovine fetal serum (Gibco 15000-044, Brazil), 100 µL/mL antibiotic-antimycotic (Sigma A5955, St. Louis, MO, USA), and 2% phytohemagglutinin (Sigma L8754, St. Louis, MO, USA). Cultures were incubated at 37 °C for 44 h under 5% CO_2_. After this incubation period, 6 µg/mL cytochalasin B (Sigma C6762, St. Louis, MO, USA) was added. After incubation, lymphocytes were harvested by centrifugation at 1200 rpm for 8 min, centrifuged again, fixed in 25:1 (*v*/*v*) methanol/acetic acid, placed on a clean microscope slide, and stained with Diff-Quik stain (Medion diagnostics; 726443. Düdingen. CH). For each participant’s blood sample, 2000 binucleated (BN) cells (1000 from each of two slides prepared from duplicate cultures) were evaluated for the presence of DNA damage indices—micronuclei (MN) using bright-field optical microscopy at 200–1000× magnification. All slides were coded for blinded analysis according to the criteria proposed by Fenech.

### 2.6. Statistical Methods

The databases were created using Epi Info 7.0, and the statistical analyses were performed with the Stata 17 program. Values reported by the laboratory as not detected or below the detection limit were considered zero in the statistical analyses. First, descriptions of participants with proportions or measures of central tendency and dispersion were used according to the observed distribution. Shapiro–Wilk test was used to explore the distribution of continuous variables. Comparisons between the three population groups were made with *x*^2^ or Kruskal–Wallis tests. Then, a detailed exploration of possible element mixtures suggested that in each mining site, there are different distributions of elements. For this reason, the following analysis was performed for each mine independently.

**Figure 2:**
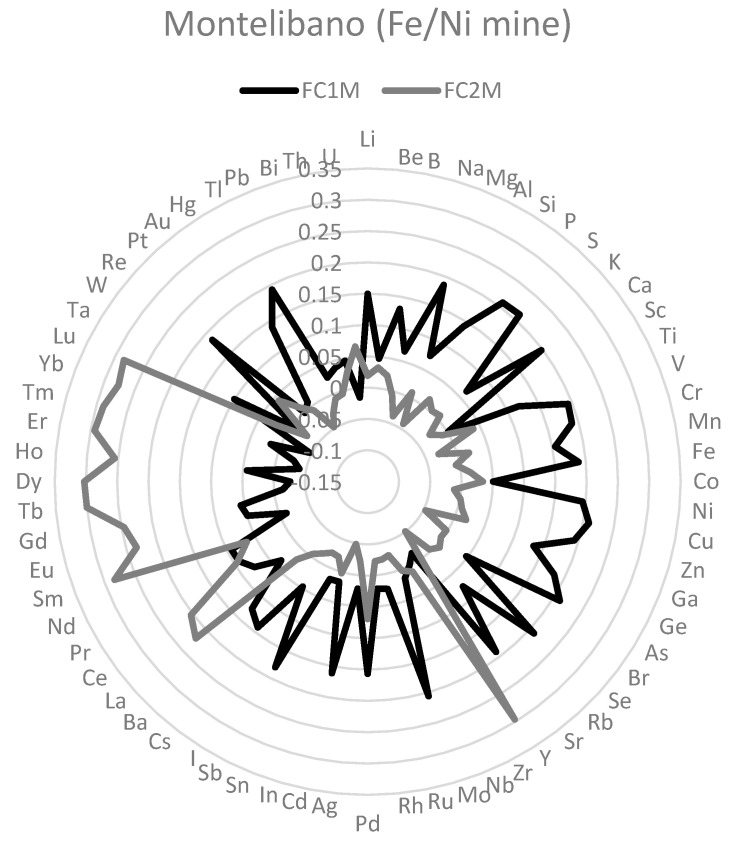
Main element mixtures in Montelibano (factor loadings).

**Figure 3:**
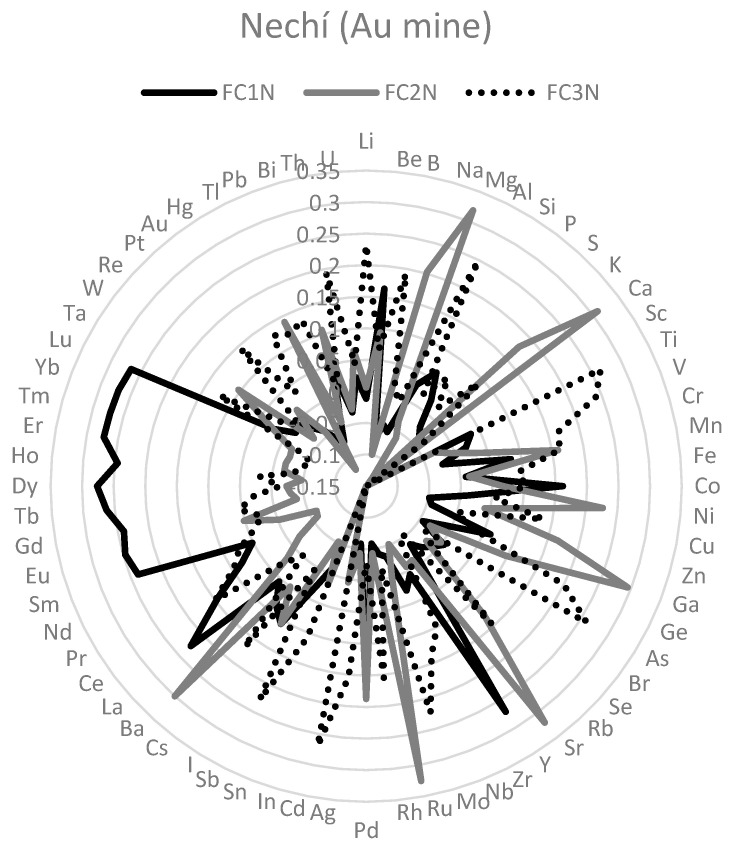
Main element mixtures in Nechí (factor loadings).

**Figure 4:**
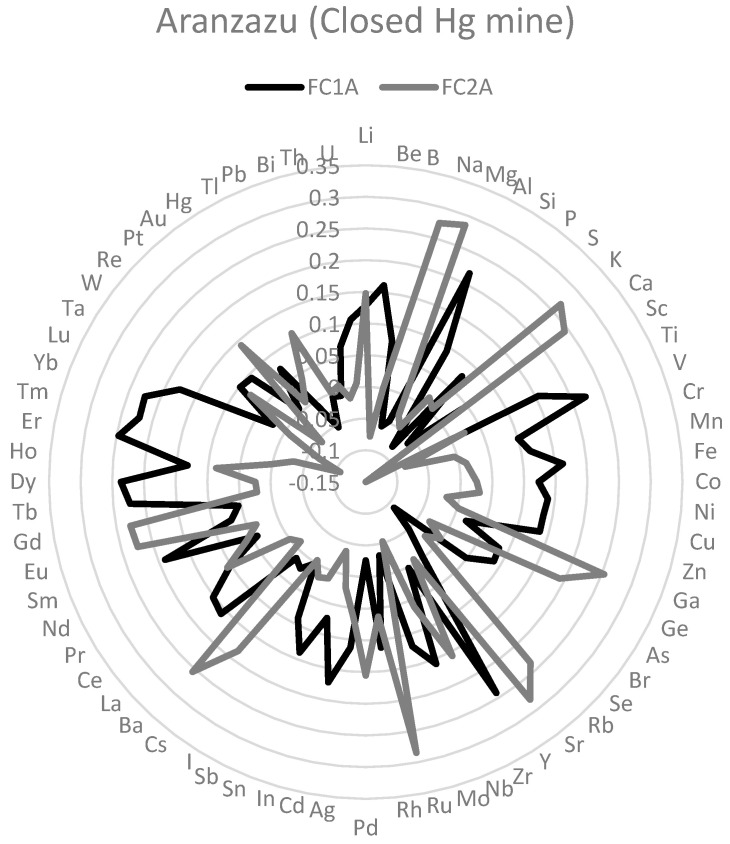
Main element mixtures in Aranzazu (factor loadings).

**Figure A1:**
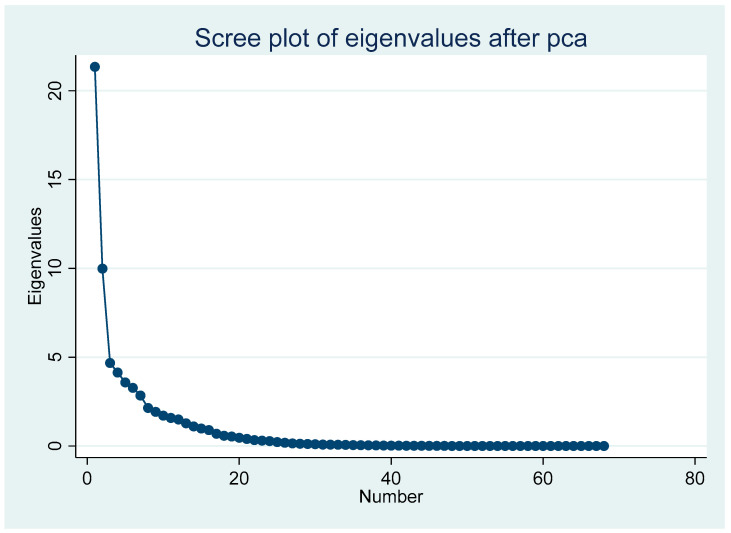
Montelibano mine. Scree plot of eigenvalues after principal component analysis.

**Figure A2:**
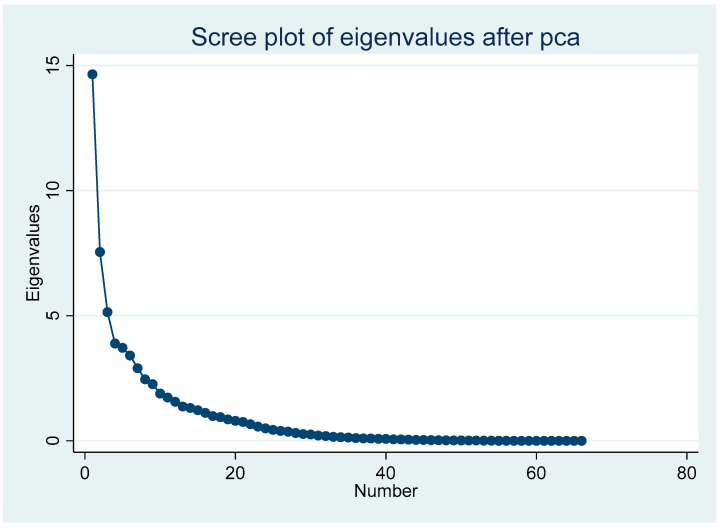
Nechí mine. Scree plot of eigenvalues after principal component analysis.

**Figure A3:**
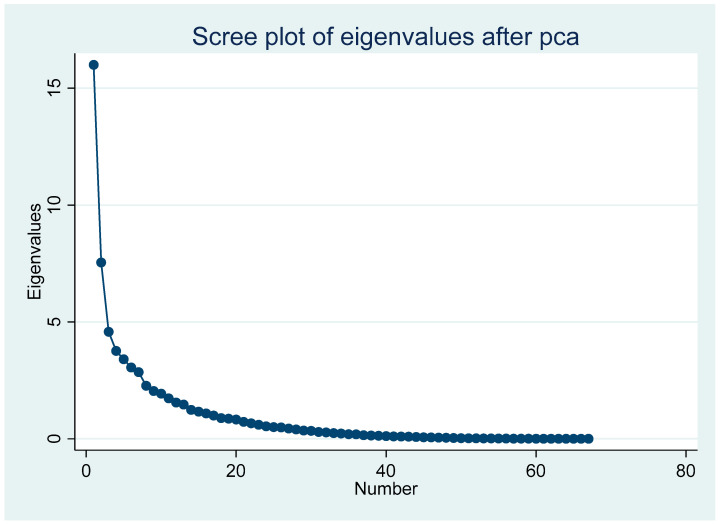
Aranzazu mine. Scree plot of eigenvalues after principal component analysis.

To identify the mixtures of elements in each mining site, principal component analyses with the 68 element concentrations in hair were carried out. It was useful because it is a mathematical model that explains covariance or correlation among element concentrations in hair in terms of unobserved latent variables [39], in this case, element mixtures. One a priori criterion to consider a factor as relevant was that they exhibited a proportional contribution higher than 5%. In Montelibano, two common factors (FC1M and FC2M) were extracted after a varimax rotation (Figure 2). With the same method, in Nechí (FC1N, FC2N, and FC3N) (see Figure 3) and Aranzazu (FC1A and FC2A) (see Figure 4), three and two factors were extracted, respectively. In Montelibano, the factors explained 46.08% of the variance, whereas, in Nechí and Aranzazu, it was 41.43% and 35.15%, respectively. Rotated components and scree plots are available in Appendix A (Table A3, Table A4 and Table A5, Figure A1, Figure A2 and Figure A3). In addition, factor scores were calculated for every element, and after these scores were normalized.

To explore the associations with the frequency of micronuclei, prevalence ratios (PR) with 95% confidence intervals (95% CI) were estimated for each mining site. They were first estimated bivariate and then multiple, using Poisson regression models with robust variance [40]. In the analysis, special care was taken with some elements that could have adverse or antagonistic effects. It was considered whether it belonged to the chemical mixtures included in the analyses; if they were not included, they were analyzed independently in each mining site. Interactions between selenium and other elements were explored in Nechí and Aranzazu because of its potential protective effect [41].

## 3. Results

### 3.1. Mean Characteristics of Participants

Information from 306 individuals was collected, but after excluding those with missing data, information from 247 individuals was analyzed. Of these, 30.77% were from Montelibano (n *=* 76), 34.01% from Nechí (n = 84), and 35.22% from Aranzazu (n = 87). It was evident that participants differed between the three locations (see Table 1). There was high participation of women, much higher in Montelibano and lower in Aranzazu. Age was also higher in Montelibano and lower in Aranzazu. There were many more married or cohabiting in Montelibano and more single in Nechí. Regarding education, it is evident that the lowest levels were found in Nechí, including a very high proportion of illiterates, which contrasts with the highest educational levels in Montelibano. As expected, there were more individuals carrying out mining-related activities in Montelibano and none in Aranzazu since the mercury mine has been closed since 1975; however, Aranzazu is where more individuals were engaged in work related to agriculture.

Cigarette consumption was higher in Aranzazu and lower in Montelibano, while alcoholic beverage consumption was higher in Montelibano and lower in Aranzazu. Regarding food consumption, it was striking that the only difference was in the consumption of fish, which was higher in Montelibano and lower in Aranzazu. In relation to the elements in the hair, it was evident that the highest concentrations are found in places where there are active mines; these include elements that, in some of their chemical forms, are carcinogenic or probably carcinogenic for humans, according to the IARC. Selenium, with its potential chelating effect, had a higher concentration in Nechí and a lower one in Aranzazu.

### 3.2. Pesticide Exposure

In relation to the quantified pesticides (Table 2), the most relevant finding is that they were detected in only a low proportion of the participants, which suggests a low number of individuals with recent exposure. In the blood samples, the following were not detected: detected aldicarb, alpha-endosulfan, betha-endosulfan, endosulfan-sulfate, malathion, hexachlorobenzene, parathion-methyl, propoxur, and carbofuran. In the urine samples, the following were not detected: aldicarb, alpha-endosulfan, betha-endosulfan, endosulfan-sulfate, malathion, hexachlorobenzene, and carbofuran. In Montelibano, more pesticides were detected, where paraoxon-methyl in blood was the most frequent, whereas, in Aranzazu, the most common was paraoxon-ethyl in blood.

### 3.3. Element Mixtures in Hair

There were very different mixtures of elements in the populations residing in the surroundings of the three mines (see Figure 2, Figure 3 and Figure 4). In the FC1M mixture in Montelibano, the most important elements were P, S, Cu, As, Se, Mo, Li, Mg, Ca, V, Cr, Mn, Fe, Ni, Zn, Ge, Sr, Pd, Cd, Sb, Re, and Tl. This factor included the elements with economic interest (Fe/Ni) and recognized toxins, such as As, Cd, Mn, Ta and Cr, and Se as potential chelators. This mixture is highly dangerous to the health of those who are exposed, as it was described previously [25,26]. FC2M included Dy, Tb, Y, Er, Tm, Lu, Sm, Yb, Ho, Gd, Eu, La, and Ce. This mixture included rare earth elements (REEs) related to modern products, such as electronics, chemicals, medical, aviation, and defense technologies, and, more recently, agriculture [42]. Some studies suggest that REEs are associated with nephrotoxic, neurotoxic, and reproductive disorders, fibrotic tissue injury, oxidative stress, pneumoconiosis, and cytotoxicity [43]. Maybe REEs are the consequence of the smelting processes in the ferronickel mine. Note that Be, Hg, and Pb have no important participation in these mixtures.

In Nechí, FC1N included Dy, Er, Tm, Yb, Y, Lu, Tb, Eu, Ho, Gd, Sm, La, Pd, Be, and Co. This mixture included less frequent elements together with Be and Co. FC2N in Nechí included Ru, Sr, Mg, Ca, Ba, Ga, Ni, Na, Pd, K, Zn, and Mn, with Ni and Mn being the most toxic mixture. FC3N in Nechí included Cd, Ti, As, Al, V, Li, Sb, Mo, Ge, Th, B, Cs, Cr, Mn, Rh, and Ag. This is the most toxic mixture in Nechí. Note that Hg, Se, and Pb have no important participation in these mixtures. FC1A in Aranzazu included Er, Y, Dy, Yb, Tb, V, Tm, Al, Sm, Lu, Cd, Fe, Be, La, Nb, Ti, and Ce. In this mixture, the most toxic elements were Cd and Be. FC2A in Aranzazu included Mg, Ru, Sr, Na, K, Ba, Ga, Ca, Rb, Gd, Eu, Ge, Cs, Zr, and Pd. This element mixture only included Mg as a recognized toxic. Note that Hg, Se, As, Cr, Mn, P, and Pb have no important participation in these mixtures.

### 3.4. Micronuclei Frequency

**Figure 5:**
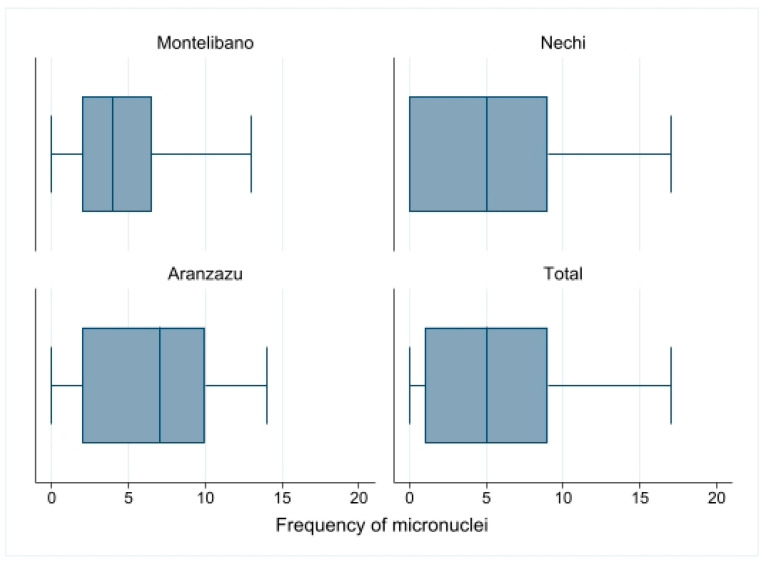
Frequency of micronuclei in the three mining contexts.

The distribution of micronuclei observed in study participants by the municipality and in total is shown in Figure 5. As can be seen, in Montelibano, the median was 4 micronuclei with 25th and 75th percentiles of 2 and 6.5; in Nechí, the median was 5 micronuclei with 25th and 75th percentiles of 0 and 9, and in Aranzazu the median was 7 micronuclei with 25th and 75th percentiles of 2 and 10. However, there were no statistically significant differences between the three mining contexts (*p* = 0.1298, Kruskal–Wallis test).

### 3.5. Bivariate Analysis

A first bivariate exploration (Table 3) of the possible associations with the number of micronuclei allowed us to identify that being a woman is protective in Montelibano, suggesting a differential occupational effect compared to men. Higher educational levels were protective, with their most noticeable effect being in Montelibano. Food consumption seems to mark routes of intake of toxic elements since the consumption of fish, white meat, and vegetables were risk factors in Montelibano, while the consumption of fruits and vegetables was protective in Nechí and Aranzazu, respectively. In relation to the mixtures of elements in hair, it was striking that no mixture showed an association in Montelibano, while in Nechí, a mixture (FC1N) with a protective effect was observed, and in Aranzazu there was a mixture (FC1A) that was a risk factor. Mercury in hair was a risk factor in Aranzazu, which was where the lowest concentration was observed among its inhabitants. Contrary to expectations, selenium in hair was a risk factor in Nechí, where the highest values of selenium were. Something similar happened with lead in hair, which was a protective factor in Nechí. Another unexpected finding is that beryllium in hair was a protective factor in Montelibano.

### 3.6. Multiple Analysis

Table 4 shows the most relevant variables associated with the number of micronuclei obtained in the multiple regression models. In Montelibano, it turned out that the consumption of fish and lead in hair were risk factors, as well as the pesticides parathion ethyl in blood and paraoxon ethyl in urine. The mixture of FC1M elements, beryllium in hair, and the consumption of vegetables and red meat were protective factors. Due to its importance, it should be clear that selenium is part of the FC1M mix.

In Nechí, the FC2N mixture (which includes Mg, Mn, and Ni), lead, and selenium were identified as risk factors. FC1N (with beryllium and very rare elements) and fruit consumption appeared as protective factors. Additionally, an interaction between lead and selenium was identified. In Aranzazu, the main risk factor is mercury, followed by nickel. Selenium and fruit consumption were identified as protective factors. A strong interaction between mercury and selenium was identified.

These findings may seem strange at first approximation but can be better understood by considering the different exposure pathways involved in each type of mine. In Montelibano, the exposure can be food and aerial, while in Nechí, it is mainly food in the context of occupational and para-occupational aerial exposure to mercury. In Aranzazu, the exposure is mainly for food.

## 4. Discussion

The most important finding of this study is the great heterogeneity in the exposure to pesticides and elements in the three contexts that combine mining and the use of pesticides in agriculture or to control vector-borne diseases. Within the complexity, it was clear that where there is recent mining activity is where more pesticides and elements were detected, and higher concentrations were observed. This is logical since mining mobilizes large amounts of rocks and soils, facilitating the entry of various elements to humans; these mainly through food and water, although the respiratory tract is likely in places with open pit mining. In fact, chemical mixtures were associated with the number of micronuclei only in Montelibano and Nechí, where there are active mines. In Aranzazu, the associations are between isolated elements or in interaction with another element.

In addition to this expected finding, selenium showed two different faces. On the one hand, selenium acted as a protective factor and, on the other, as a risk factor framed in the context of statistical interaction. This seems to be strong evidence of the “hormetic response” [44], in which selenium at low doses is beneficial (Aranzazu case) or acts synergistically with other chemicals (Nechí case) when it is in high concentrations, increasing the adverse effect. In the case of Montelibano, the presence of selenium in a mixture with many toxic elements seems to be related to the mitigation of the adverse effect. Note that the observed concentrations of selenium in hair in Montelibano were intermediate between Nechí and Aranzazu. To the best of our knowledge, this is the first time that the “hormetic effect” of selenium in humans has been reported under non-experimental conditions. Hormetic dose response has been described for several substances in different animal species [45]. Identifying the “hormetic effect” of a substance, such as selenium, allows us to understand the contradictory effects observed in several studies. Unfortunately, it is not easy to define the adequate dose related to only beneficial effects.

Reduction of exposure to toxic metals and metalloids using Se is a priority among susceptible populations, such as children and pregnant and lactating women. In general, consumption of selenium-rich foods can be part of interventions to avoid the adverse effects of contaminants, such as Hg [46]. The intake of Se can come from foods that, by definition, have higher concentrations, such as nuts, or from the consumption of artificially enriched foods. Another option is to apply Se to crops, such as rice, reducing Hg accumulation in plants [47]. However, special care must be taken in places where the geology includes selenium in high concentrations because synergistic adverse effects, such as those observed in Nechí in this study, may occur. Knowing the history is important because, in the 1626, fray Pedro Simon described the toxicity of Se in corn and other vegetables that grew in specific places; poisoned animals and humans lost their hair and had dermal lesions [48]. In consequence, although there are some regional studies [49], Colombia needs to improve studies on Se in soils, especially in places where there are mining activities.

In Aranzazu, there is a health issue that must be pointed out due to the possible implications it has on the results, and that, unfortunately, could not be addressed in the present study. Among the inhabitants of Aranzazu, endogamy has been common for several generations; in fact, the occurrence of neuropsychiatric disorders, such as mood bipolar disorder, is so high that studies have been carried out that report its association with the genetic factor [50]. In this case, the question arises as to whether these genetic changes have an influence on the number of micronuclei observed, such as that of the other mining sites.

This study should be interpreted considering the limitations of the study. First, the population was not representative, so the findings should be understood as exploratory of important environmental health problems. Interest in the study was the main motivation of the participants, which explains the greater participation of women; men tend to be more cautious in places where there are illegal armed groups, as occurred in Montelibano and Nechí. Colombia is one of the countries with more socio-ecological conflicts [51] and where more assassinations of environmental leaders occur [52], which is evidence of the difficulties of carrying out environmental health studies in places with the greatest contamination.

Perhaps no pesticides were found because it is easier to find them in those who directly carry out fumigation activities, which by tradition is an activity associated with men. Given that the miners could be the ones most exposed to elements, it is possible that their low participation has the effect that their results are higher than reported. An important issue to note is that, due to the high number of elements included, several of these do not have certified standards. However, due to the exploratory and novel nature of the study approach, it was decided to include all the results. If there was a measurement error, we have no evidence that it was differential.

In conclusion, the results show the complex exposures that occur in mining contexts where there are also pesticides used in agricultural activities or to control vector-borne diseases. Each place is so different from the others that it is difficult to extrapolate the findings to other similar areas. In the Colombian case, it is a difficult task because the data on the use of pesticides do not have adequate information; it is even more complicated in places where illegal activities are carried out, such as coca, marijuana, or poppy cultivation [15]. In addition, soil geochemistry data is scarce, and much of what exists is only available to mining companies that collect data as part of their economic activities. For this reason, studies such as this one are essential to understand the effects of large extractive activities, especially if they were carried out before and after starting work.

Additionally, this study identified the region where the open pit ferronickel mine is located as the most polluted and which demands urgent attention from the environmental and health authorities. Since this may be happening at other large open pit mines, it is important to perform similar studies in regions with coal in La Guajira [53] and Cesar departments [54] and gold mining in La Colosa in Cajamarca, Tolima [55]. Periodic monitoring of environmental agents, mainly in the most polluted sites, is essential to have adequate vigilance of the potential adverse effects associated with chemical agents. Without a doubt, it is very important that Colombian authorities prioritize toxic chemicals because there is an exaggerated use of pesticides and many extractive activities with a high impact on the environment.

## Figures and Tables

**Table 1:** Mean characteristics of individuals participating in the study (n = 247).

Site (Minerals)	Montelibano (Fe/Ni)	Nechí (Au)	Aranzazu (Closed Hg)	
Variables	(n = 76) %/Median (Min–Max)	(n = 84) %/Median (Min–Max)	(n = 87) %/Median (Min–Max)	*p* Value
Sex (female)	80.26	72.62	60.92	0.023
Age (years)	54 (21–81)	48 (21–83)	40 (19–83)	<0.001 ^a^
Civil status				
Married/Free union	85.53	65.48	70.11	0.029
Divorced/Widower	2.63	7.14	10.34	
Single	11.84	27.38	19.54	
Education				
Illiteracy	2.63	11.90	5.75	<0.001
Elementary (partial or full)	13.16	39.29	39.08	
Secondary (partial or full)	31.58	22.62	37.93	
Technic (partial or full)	35.53	19.05	4.60	
University (partial or full)	17.11	7.14	12.64	
Occupation				
In mining activities	52.63	44.05	0	<0.001
In agricultural activities	6.58	22.62	45.98	<0.001
Cigarette consumption (any moment)	21.05	39.29	43.68	0.007
Alcohol consumption (any moment)	61.84	44.05	40.23	0.015
Food consumption				
Fish	97.37	91.67	71.26	<0.001
Canned food	56.58	48.81	60.92	0.274
White meat	97.37	92.86	96.55	0.328
Red meat	94.74	94.05	96.55	0.734
Fruits	96.05	94.05	94.25	0.825
Vegetables	98.68	97.62	94.25	0.242
Carcinogenic elements * (IARC 1)				
Arsenic	0.14 (0.05–4.28)	0.18 (0.06–1.64)	0.12 (0.05–0.36)	<0.001 ^a^
Beryllium	0 (0–16.06)	0 (0–0.07)	0 (0–0.02)	<0.001 ^a^
Cadmium	0.05 (0–1.69)	0.04 (0.01–0.48)	0.05 (0.01–0.94)	0.395 ^a^
Chromium	0.51 (0.18–19.06)	0.33 (0.18–7.25)	0.33 (0.20–3.53)	<0.001 ^a^
Strontium	2.09 (0.1–55.42)	3.49 (0.33–46.21)	2.97 (0.19–46.13)	0.011 ^a^
Nickel	0.45 (0.05–17.77)	0.25 (0.05–2.07)	0.14 (0.02–3.92)	<0.001 ^a^
Thorium	0 (0–0.27)	0 (0–0.04)	0 (0–0.01)	<0.001 ^a^
Probably carcinogenic * (IARC 2A)				
Antimony	0.03 (0.00–0.35)	0.02 (0.00–0.35)	0.01 (0.00–0.29)	0.040 ^a^
Cobalt	0.04 (0.00–4.46)	0.03 (0.01–1.14)	0.01 (0.00–0.13)	<0.001 ^a^
Indium	0 (0–0.002)	0	0 (0–0.004)	0.3484
Magnesium	77.90 (12.97–1449)	105.81 (19.44–2131)	49.73 (9.51–608.74)	<0.001 ^a^
Manganese	1.66 (0.06–92.63)	3.09 (0.15–46.54)	0.80 (0.10–11.50)	<0.001 ^a^
Silica	0 (0 -7598)	0 (0–4495)	1201 (0–3913)	<0.001 ^a^
Possibly carcinogenic * (IARC 2B)				
Mercury	1.89 (0.06–12.29)	2.48 (0.17–17.14)	0.08 (0–1.63)	<0.001 ^a^
Molybdenum	0.05 (0.01–1.40)	0.06 (0.02–0.36)	0.04 (0.02–0.24)	<0.001 ^a^
Lead	0.92 (0.04–89.01)	0.64 (0.08–75.17)	0.55 (0.06–163.37)	0.761 ^a^
Potassium	1.83 (0–155.47)	3.98 (0–106.10)	1.08 (0–129.28)	<0.001 ^a^
Sodium	3.12 (0.47–238.26)	3.94 (0.49–701.25)	1.44 (0.30–379.54)	<0.001 ^a^
Titanium	0.13 (0–3.16)	0.24 (0.09–1.24)	0.18 (0.08–0.60)	<0.001 ^a^
Vanadium	0.05 (0.00–1.41)	0.12 (0.03–0.85)	0.05 (0.02–0.45)	<0.001 ^a^
Other elements				
Phosphorus	219 (66–2883)	218 (111–342)	205 (148–297)	0.114 ^a^
Selenium	1.35 (0.23–14.70)	1.40 (0.46–60.69)	1.01 (0.41–1.44)	<0.001 ^a^

* Classification of the International Agency for Research on Cancer. ^a^ Kruskal-Wallis test.

**Table 2:** Concentrations of selected pesticides in blood and urine among inhabitants near three mines in Colombia.

Montelibano (Fe/Ni Open-Pit Mine)			Concentrations (ppm)					
		Positives		Percentiles				
Pesticide	Sample	%	Min	25	90	95	99	Max
Paraoxon-methyl	Blood	27.55	ND	ND	0.24	0.29	37.26	37.26
Parathion-ethyl	Blood	1.02	ND	ND	ND	ND	ND	1.02
Paraoxon-ethyl	Urine	2.04	ND	ND	ND	ND	0.06	0.06
Parathion-ethyl	Urine	2.04	ND	ND	ND	ND	0.07	0.07
Parathion-methyl	Urine	2.04	ND	ND	ND	ND	0.07	0.07
Propoxur	Urine	2.04	ND	ND	ND	ND	0.13	0.44
**Nechí** (Au fluvial mines)			**Concentrations (ppm)**					
		**Positives**		**Percentiles**				
**Pesticide**	**Sample**	**%**	**Min**	**25**	**90**	**95**	**99**	**Max**
Paraoxon-ethyl	Blood	0.99	ND	ND	ND	ND	ND	0.75
Parathion-ethyl	Urine	0.99	ND	ND	ND	ND	ND	0.05
**Aranzazu** (Hg closed mine)			**Concentrations (ppm)**					
		**Positives**		**Percentiles**				
**Pesticide**	**Sample**	**%**	**Min**	**25**	**90**	**95**	**99**	**Max**
Paraoxon-ethyl	Blood	18.81	ND	ND	0.08	0.09	0.10	0.13
Paraoxon-methyl	Urine	0.99	ND	ND	ND	ND	ND	0.38

ND: Not Detectable.

**Table 3:** Crude prevalence ratios (PR) between pesticide and element exposure and micronuclei occurrence.

Site	Montelibano (n = 76)		Nechí (n = 84)		Aranzazu (n = 87)	
(Type of Mine)	Fe/Ni		Au		Closed Hg	
Variables	PR	95% CI	PR	95% CI	PR	95% CI
Sex (female)	0.68	0.47–0.99	0.82	0.56–1.21	0.86	0.63–1.17
Age (years)	1.01	0.99–1.02	1.00	0.99–1.01	1.00	0.99–1.01
Civil status						
Married/Free union	1		1		1	
Divorced/Widower	1.26	0.51–3.11	1.31	0.64–2.68	1.14	0.68–1.91
Single	1.17	0.68–2.02	1.26	0.85 -1.87	1.01	0.70–1.45
Education						
Illiteracy	1		1		1	
Elementary (partial or full)	0.38	0.24–0.60	1.02	0.56–1.86	0.63	0.42–0.94
Secondary (partial or full)	0.48	0.34–0.66	0.85	0.46 -1.59	0.67	0.43–1.03
Technic (partial or full)	0.41	0.31–0.53	1.00	0.53–1.89	0.78	0.40–1.54
University (partial or full)	0.45	0.31–0.64	1.29	0.65–2.55	0.78	0.50–1.22
Occupation						
In mining activities	0.90	0.66–1.23	1.12	0.77–1.64	NA	
In agricultural activities	1.13	0.71–1.79	0.58	0.33–1.02	0.99	0.72–1.35
Consumption (any moment)						
Cigarette	1.33	0.94–1.87	0.88	0.58–1.33	1.15	0.84–1.57
Alcohol	0.81	0.59–1.12	1.18	0.81–1.71	0.86	0.62–1.17
Food consumption						
Fish	2.27	1.11–4.64	1.74	0.53–5.74	1.33	0.91–1.97
Canned food	1.14	0.84–1.54	1.44	0.99–2.08	1.09	0.78–1.54
White meat	1.29	1.00–1.66	1.93	0.80–4.65	1.05	0.67–1.64
Red meat	0.89	0.62–1.26	1.34	0.45–4.01	0.84	0.35–2.04
Fruits	1.12	0.38–3.30	0.48	0.32–0.71	0.73	0.52–1.01
Vegetables	1.50	1.28–1.75	0.52	0.19–1.39	0.75	0.58–0.96
Pesticides (ppb)						
Paraoxon-methyl in blood	1.00	0.99–1.01				
Paraoxon-ethyl in blood					18.43	0.71–476.02
Element mixtures in hair						
FC1	0.97	0.94–1.00	0.90	0.83–0.98	1.04	1.00–1.07
FC2	0.98	0.94–1.03	1.04	0.99–1.09	1.02	0.99–1.05
FC3	NA		0.96	0.90–1.03	NA	
Selected elements in hair						
Mercury	0.97	0.93–1.02	0.98	0.92–1.03	1.76	1.06–2.93
Selenium	NA		1.02	1.01–1.02	0.58	0.25–1.34
Lead	1.01	1.00–1.01	0.94	0.89–0.98	1.00	0.99–1.00
Beryllium	0.77	0.66–0.89	NA		NA	

NA: Not Applicable.

**Table 4:** Adjusted prevalence ratios (adj. PR) between pesticide and element exposure and micronuclei occurrence.

Site	Montelibano (n = 76)		Nechí (n = 84)		Aranzazu (n = 87)	
(Type of Mine)	(Open-Pit Fe/Ni)		(Fluvial Au)		(Closed Hg)	
Variables	Adj. PR	95% CI	Adj. PR	95% CI	Adj. PR	95% CI
Pesticides (ppb)						
Parathion ethyl (blood)	0.04	0.01–0.14				
Paraoxon ethyl (urine)	0.01	0.00–0.04				
Element mixtures in hair (scores)						
Factor 1	0.97	0.94–0.99	0.88	0.79–0.98		
Factor 2			1.05	1.00–1.09		
Hair concentration (ppm)						
Beryllium	0.67	0.59–0.76				
Lead	1.01	1.00–1.01	1.37	1.06–1.77		
Mercury					36.02	2.69–380.55
Selenium			1.05	1.03–1.08	0.86	0.32–2.33
Nickel					1.28	1.13–1.45
Interactions						
Selenium X Lead			0.78	0.64–0.95		
Selenium X Mercury					0.11	0.02–0.77
Diet consumption						
Fish	4.73	3.94–5.68				
Vegetables	0.47	0.38–0.59				
Red meat	0.67	0.54–0.83				
Fruits			0.55	0.38–0.80	0.66	0.46–0.95

## Data Availability

The data presented in this study are available on request from the corresponding author.

## Appendix A

Validation data of pesticide analyses—blood/urine LC/Ms-Ms QUECHERS extraction; Main element mixtures in each mining site obtained with principal component analysis.

**Table A1:** Validation data—blood LC/Ms-Ms QuEChERS extraction.

Pesticide	Lineal Range (ppb)	Detection Limit (ppb)	Quantification Limit (ppb)
Aldicarb (ppb)	0.075 to 15.00	0.01	0.075
Propoxur (ppb)	0.075 to 15.00	0.01	0.075
Carbofuran (ppb)	0.075 to 15.00	0.01	0.075
α-endosulfan (ppb)	5.00 to 50.00	1.00	5.00
β-endosulfan (ppb)	5.00 to 50.00	1.00	5.00
Endosulfan sulfate (ppb)	2.00 to 75.00	0.05	2.00
Malathion (ppb)	0.075 to 15.00	0.01	0.075
hexachlorobenzene (ppb)	0.125 to 75.00	0.025	0.125
ethyl paraoxon (ppb)	0.075 to 15.00	0.01	0.075
methyl paraoxon (ppb)	0.075 to 15.00	0.01	0.075
ethyl parathion (ppb)	0.075 to 15.00	0.01	0.075
methyl parathion (ppb)	0.075 to 15.00	0.01	0.075

**Table A2:** Validation data—urine LC/Ms-Ms QuEChERS extraction.

Pesticide	Lineal Range (ppb)	Detection Limit (ppb)	Quantification Limit (ppb)
Aldicarb (ppb)	0.075 to 15.00	0.01	0.075
Propoxur (ppb)	0.075 to 15.00	0.01	0.075
Carbofuran (ppb)	0.075 to 15.00	0.01	0.075
α-endosulfan (ppb)	5.00 to 50.00	1.00	5.00
β-endosulfan (ppb)	5.00 to 50.00	1.00	5.00
Endosulfan sulfate (ppb)	2.00 to 75.00	0.05	2.00
Malathion (ppb)	0.075 to 15.00	0.01	0.075
hexachlorobenzene (ppb)	0.125 to 75.00	0.025	0.125
ethyl paraoxon (ppb)	0.075 to 15.00	0.01	0.075
methyl paraoxon (ppb)	0.075 to 15.00	0.01	0.075
ethyl parathion (ppb)	0.075 to 15.00	0.01	0.075
methyl parathion (ppb)	0.075 to 15.00	0.01	0.075

**Table A3:** Montelibano mine. Rotated components.

Variable	Comp1	Comp2	Unexplained
Li7ppm	0.1500	0.0191	0.52
Be9ppm	0.0473	0.0327	0.9327
B11ppm	0.1305	0.0220	0.63
Na23ppm	0.0662	−0.0063	0.9131
Mg24ppm	0.1870	−0.0380	0.3187
Al27ppm	0.0742	0.0091	0.8829
Si28ppm	0.1413	−0.0416	0.6132
P31ppm	0.2082	0.0145	0.09689
S34ppm	0.2107	0.0026	0.09373
K39ppm	0.0639	0.0067	0.9136
Ca44ppm	0.1978	−0.0258	0.2299
Sc45ppm	0.0012	−0.0089	0.9992
Ti47ppm	0.1200	0.0388	0.6604
V51ppm	0.1932	−0.0285	0.2674
Cr52ppm	0.1895	0.0186	0.2429
Mn55ppm	0.1549	−0.0064	0.5178
Fe57ppm	0.1885	0.0145	0.2573
Co59ppm	0.0504	0.0342	0.9244
Ni60ppm	0.1947	−0.0113	0.2421
Cu65ppm	0.2097	0.0007	0.1052
Zn66ppm	0.1931	0.0067	0.2328
Ga69ppm	0.1342	0.0188	0.6137
Ge74ppm	0.1828	−0.0469	0.3514
As75ppm	0.2112	−0.0023	0.09576
Br79ppm	0.0475	−0.0033	0.9551
Se82ppm	0.2097	0.0057	0.09745
Rb85ppm	0.0761	−0.0035	0.8838
Sr88ppm	0.1905	−0.0503	0.2963
Y89ppm	−0.0160	0.2961	0.06107
Zr90ppm	−0.0002	0.0105	0.9988
Nb93ppm	0.0153	0.0024	0.9949
Mo98ppm	0.2059	−0.0278	0.1661
Ru102ppm	0.0243	−0.0237	0.9856
Rh103ppm	0.0215	−0.0223	0.9882
Pd105ppm	0.1568	0.0694	0.3767
Ag107ppm	0.0215	−0.0223	0.9882
Cd114ppm	0.1610	−0.0487	0.4974
In115ppm	0.0162	0.0020	0.9944
Sn118ppm	0.0167	−0.0215	0.9916
Sb121ppm	0.1809	−0.0231	0.3559
I127ppm	0.0471	−0.0154	0.957
Cs133ppm	0.1414	−0.0053	0.5982
Ba137ppm	0.1245	0.0183	0.6661
La139ppm	0.0366	0.2215	0.3809
Ce140ppm	0.0761	0.2036	0.3263
Pr141ppm	0.0914	0.0952	0.6742
Nd143ppm	0.0930	0.0656	0.7374
Sm147ppm	−0.0111	0.2836	0.1337
Eu153ppm	0.0492	0.2336	0.2762
Gd157ppm	0.0561	0.2459	0.1821
Tb159ppm	−0.0142	0.3002	0.03225
Dy163ppm	−0.0265	0.3027	0.03045
Ho165ppm	0.0436	0.2556	0.1711
Er166ppm	−0.0390	0.2938	0.09388
Tm169ppm	−0.0274	0.2887	0.1194
Yb172ppm	0.0159	0.2768	0.1242
Lu175ppm	−0.0468	0.2851	0.1482
Ta181ppm	0.1006	0.0208	0.7759
W182ppm	−0.0107	−0.0288	0.9866
Re185ppm	0.1857	0.0421	0.2286
Pt194ppm	−0.0017	0.0101	0.9989
Au197ppm	0.0089	−0.0077	0.9982
Hg202ppm	0.1397	−0.0477	0.621
Tl205ppm	0.1923	−0.0274	0.2741
Pb208ppm	0.0280	−0.0081	0.9848
Bi209ppm	0.0389	−0.0043	0.97
Th232ppm	0.0463	0.0276	0.9398
U238ppm	−0.0153	0.0667	0.9529
**Component rotation matrix**			
Comp1	0.9545	0.2982	
Comp2	−0.2982	0.9545	

**Table A4:** Nechí mine. Rotated components.

Variable	Comp1	Comp2	Comp3	Unexplained
Li7ppm	−0.0115	0.0063	0.2322	0.668
Be9ppm	0.1641	0.0943	0.0676	0.428
B11ppm	−0.0730	−0.0993	0.1918	0.6905
Na23ppm	−0.0492	0.2026	0.0141	0.6789
Mg24ppm	−0.0579	0.3185	−0.0070	0.2158
Al27ppm	0.0292	−0.0349	0.2480	0.5471
Si28ppm	0.0614	−0.0603	0.0216	0.9232
P31ppm	0.0210	−0.1330	0.0652	0.8326
S34ppm	−0.0232	−0.1452	0.0180	0.8187
K39ppm	−0.0366	0.1778	0.0907	0.7146
Ca44ppm	−0.0331	0.3095	−0.0346	0.256
Ti47ppm	0.0354	−0.0302	0.2649	0.4755
V51ppm	0.0194	−0.0054	0.2402	0.5968
Cr52ppm	−0.0241	0.0426	0.1676	0.8227
Mn55ppm	0.0832	0.1594	0.1615	0.4053
Fe57ppm	0.0088	0.0143	0.0925	0.9365
Co59ppm	0.1617	0.0669	0.1027	0.4149
Ni60ppm	0.0119	0.2265	0.0548	0.5576
Cu65ppm	−0.0485	0.0411	0.1353	0.8828
Zn66ppm	−0.0424	0.1659	−0.0004	0.7825
Ga69ppm	0.0612	0.2940	0.0238	0.2001
Ge74ppm	−0.0107	0.0976	0.2001	0.6738
As75ppm	−0.0443	−0.0311	0.2606	0.6023
Br79ppm	0.0044	−0.0016	−0.0314	0.9943
Se82ppm	−0.0081	−0.0209	−0.0087	0.9942
Rb85ppm	−0.0186	0.1297	0.1449	0.7437
Sr88ppm	−0.0344	0.3195	−0.0553	0.1947
Y89ppm	0.2702	−0.0120	−0.0440	0.1154
Zr90ppm	0.0062	−0.0070	0.1009	0.9305
Nb93ppm	0.0274	−0.0513	0.1370	0.8336
Mo98ppm	−0.0339	−0.0011	0.2230	0.7128
Ru102ppm	−0.0402	0.3249	−0.0172	0.1875
Rh103ppm	−0.0585	−0.0429	0.1548	0.8387
Pd105ppm	0.1690	0.1871	−0.0274	0.2792
Ag107ppm	−0.0585	−0.0429	0.1548	0.8387
Cd114ppm	−0.0324	0.0052	0.2684	0.5776
Sn118ppm	−0.0175	0.0126	0.1077	0.9321
Sb121ppm	0.0264	−0.0518	0.2278	0.6098
I127ppm	0.1067	0.1061	0.0060	0.7229
Cs133ppm	0.0865	0.0496	0.1696	0.5898
Ba137ppm	0.0545	0.3004	−0.0011	0.2019
La139ppm	0.2261	0.0191	0.0360	0.2558
Ce140ppm	0.1444	−0.0180	0.1492	0.4578
Pr141ppm	0.0817	−0.0595	0.0962	0.7948
Nd143ppm	0.0515	−0.0620	0.0744	0.8882
Sm147ppm	0.2375	−0.0029	0.0403	0.1947
Eu153ppm	0.2467	0.0513	0.0218	0.107
Gd157ppm	0.2404	−0.0383	0.0542	0.1628
Tb159ppm	0.2629	−0.0297	0.0305	0.05878
Dy163ppm	0.2765	−0.0237	−0.0136	0.03806
Ho165ppm	0.2456	−0.0470	0.0194	0.1984
Er166ppm	0.2728	−0.0181	−0.0349	0.09089
Tm169ppm	0.2717	−0.0177	−0.0377	0.1013
Yb172ppm	0.2706	−0.0207	−0.0560	0.13
Lu175ppm	0.2661	−0.0185	−0.0436	0.1461
Ta181ppm	0.0219	0.0252	0.1231	0.8716
W182ppm	−0.0100	0.1034	0.0185	0.916
Re185ppm	−0.0068	−0.0379	−0.0067	0.9866
Pt194ppm	−0.0261	0.0131	0.1446	0.8793
Au197ppm	−0.0408	−0.0434	0.0348	0.9597
Hg202ppm	−0.0631	−0.1192	0.1304	0.7625
Tl205ppm	−0.0717	0.1403	0.1189	0.7638
Pb208ppm	−0.0367	−0.0415	0.1321	0.885
Bi209ppm	0.0371	0.1073	0.0109	0.8753
Th232ppm	−0.0311	−0.0263	0.1933	0.7794
U238ppm	0.0228	0.0653	0.0465	0.9332
**Component rotation matrix**				
Comp1	0.9102	0.2221	0.3497	
Comp2	−0.1539	0.9650	−0.2122	
Comp3	−0.3845	0.1393	0.9125	

**Table A5:** Aranzazu (closed) mine. Rotated components.

Variable	Comp1	Comp2	Unexplained
Li7ppm	0.1282	0.1476	0.4032
Be9ppm	0.1615	−0.0780	0.6992
B11ppm	0.0763	0.0005	0.9241
Na23ppm	−0.0588	0.2748	0.2883
Mg24ppm	−0.0468	0.2847	0.2233
Al27ppm	0.2177	−0.0328	0.4314
Si28ppm	0.0929	−0.0485	0.8996
P31ppm	−0.0800	0.0166	0.9247
S34ppm	0.0755	0.0064	0.9215
K39ppm	−0.0603	0.2665	0.3328
Ca44ppm	0.0432	0.2435	0.2644
Ti47ppm	0.1547	0.0238	0.6532
V51ppm	0.2220	−0.0831	0.4365
Cr52ppm	0.1002	−0.0045	0.8731
Mn55ppm	0.1143	0.0114	0.8184
Fe57ppm	0.1626	0.0175	0.6304
Co59ppm	0.1233	0.0255	0.77
Ni60ppm	0.1389	0.0307	0.7051
Cu65ppm	0.1355	−0.0199	0.7793
Zn66ppm	0.1358	0.0027	0.7577
Ga69ppm	0.0198	0.2537	0.2743
Ge74ppm	0.0849	0.1918	0.3856
As75ppm	0.0877	−0.0314	0.912
Br79ppm	0.0488	−0.0019	0.9698
Se82ppm	−0.0894	−0.0231	0.8737
Rb85ppm	−0.0483	0.2360	0.4736
Sr88ppm	0.0024	0.2806	0.1626
Y89ppm	0.2415	−0.0064	0.2545
Zr90ppm	0.0019	0.1568	0.738
Nb93ppm	0.1582	0.0610	0.5575
Mo98ppm	0.1197	−0.0525	0.8358
Ru102ppm	−0.0322	0.2846	0.2052
Rh103ppm	0.1125	0.0636	0.735
Pd105ppm	−0.0263	0.1554	0.7689
Ag107ppm	0.1125	0.0636	0.735
Cd114ppm	0.1717	0.0207	0.5838
In115ppm	0.0733	−0.0367	0.9379
Sn118ppm	0.1385	0.0131	0.7344
Sb121ppm	0.0901	0.0179	0.8783
I127ppm	0.0012	−0.0047	0.9998
Cs133ppm	0.0212	0.1844	0.6031
Ba137ppm	0.0124	0.2555	0.2828
La139ppm	0.1589	−0.0109	0.6848
Ce140ppm	0.1520	−0.0003	0.7003
Pr141ppm	0.1014	0.1068	0.6583
Nd143ppm	0.0410	0.0606	0.9192
Sm147ppm	0.1897	0.0352	0.4652
Eu153ppm	0.0707	0.2241	0.276
Gd157ppm	0.0545	0.2279	0.312
Tb159ppm	0.2235	0.0222	0.306
Dy163ppm	0.2364	0.0241	0.2221
Ho165ppm	0.1325	0.0871	0.5982
Er166ppm	0.2470	0.0025	0.2027
Tm169ppm	0.2213	−0.0317	0.4104
Yb172ppm	0.2237	−0.1070	0.4231
Lu175ppm	0.1777	−0.0688	0.6391
Ta181ppm	0.0220	−0.0088	0.9945
W182ppm	0.0982	0.0779	0.7488
Re185ppm	0.0937	−0.0567	0.8952
Pt194ppm	0.0062	0.1416	0.7806
Au197ppm	0.0734	0.0080	0.9246
Hg202ppm	−0.0141	0.0413	0.9841
Tl205ppm	−0.0535	0.1120	0.8788
Pb208ppm	−0.0062	0.0018	0.9996
Bi209ppm	−0.0086	0.0077	0.999
Th232ppm	0.0674	−0.0158	0.947
U238ppm	0.1072	0.0069	0.8444
**Component rotation matrix**			
Comp1	0.8020		
Comp2	0.5974	−0.8020	
